# A Survey on Reinforcement Learning Methods in Bionic Underwater Robots

**DOI:** 10.3390/biomimetics8020168

**Published:** 2023-04-20

**Authors:** Ru Tong, Yukai Feng, Jian Wang, Zhengxing Wu, Min Tan, Junzhi Yu

**Affiliations:** 1 State Key Laboratory of Management and Control for Complex Systems, Institute of Automation, Chinese Academy of Sciences, Beijing 100190, China; 2 School of Artificial Intelligence, University of Chinese Academy of Sciences, Beijing 100049, China; 3 State Key Laboratory for Turbulence and Complex Systems, Department of Advanced Manufacturing and Robotics, College of Engineering, Peking University, Beijing 100871, China

**Keywords:** bionic underwater robot, reinforcement learning, robotic fish, intelligent control

## Abstract

Bionic robots possess inherent advantages for underwater operations, and research on motion control and intelligent decision making has expanded their application scope. In recent years, the application of reinforcement learning algorithms in the field of bionic underwater robots has gained considerable attention, and continues to grow. In this paper, we present a comprehensive survey of the accomplishments of reinforcement learning algorithms in the field of bionic underwater robots. Firstly, we classify existing reinforcement learning methods and introduce control tasks and decision making tasks based on the composition of bionic underwater robots. We further discuss the advantages and challenges of reinforcement learning for bionic robots in underwater environments. Secondly, we review the establishment of existing reinforcement learning algorithms for bionic underwater robots from different task perspectives. Thirdly, we explore the existing training and deployment solutions of reinforcement learning algorithms for bionic underwater robots, focusing on the challenges posed by complex underwater environments and underactuated bionic robots. Finally, the limitations and future development directions of reinforcement learning in the field of bionic underwater robots are discussed. This survey provides a foundation for exploring reinforcement learning control and decision making methods for bionic underwater robots, and provides insights for future research.

## 1. Introduction

With the development of technology, the demand for developing underwater resources is increasing. The application of bionic underwater robots, characterized by low power consumption, high maneuverability, and environmental friendliness, has become a rapidly developing research field. In the past decades, research into bionic underwater robots, by imitating the shape, swimming patterns, and behaviors of fish, has achieved promising results concerning propulsion mechanisms [1,2], bionic propulsion design [3,4,5,6,7,8], high performance optimization [9,10,11,12], and other aspects. Furthermore, extended research involving motion control, target tracking, and underwater navigation has been conducted for bionic underwater robots. In 2015, Ren et al. [13] developed a data-driven motion control framework for a two-jointed robotic fish, and achieved the desired motion. Wang et al. [14] explored the path-following control for a bionic underwater vehicle propelled by a ribbon fin. Additional, Travis et al. [15] investigated a visual and goal-conditioned navigation strategy for autonomous underwater vehicles to conduct underwater navigation tasks without any prior map training. However, the control of bionic underwater robots still faces two challenges. First, bionic robots are typically underactuated systems with highly nonlinear dynamics, and the hydrodynamic analysis of their dynamics modeling is complex [16]. Second, the underwater environment is much more unstable, and not only is it susceptible to interference with the swing of the robot’s body and fin, it is also not easily modeled [17]. Under the disturbances of the aquatic environment and the influence of strong nonlinearities, achieving satisfactory control performance for bionic robots remains challenging [18].

In 2015, reinforcement learning (RL) demonstrated impressive results in Go AI [19,20]. Since then, the performance and computational efficiency of reinforcement learning algorithms have been continuously iterated and optimized, making it a promising approach for intelligent control in robotics. Reinforcement learning promotes the natural evolution of control policies with minimal effort [21]. Based on reinforcement learning, robots interact with the environment and update their control policies, learning to solve sequential decision making problems. Reinforcement learning is a dynamic learning process that continuously interacts with the environment to obtain policy optimization, making it suitable for robot tasks that require solving sequential decision making problems.

There have been numerous studies on the application of reinforcement learning in robot control. In 2017, Cui et al. proposed an actor–critic network (AC) and RL-based trajectory tracking control method for an AUV that considers external disturbances, control input nonlinearities, and model uncertainties [22]. In 2020, Lee et al. studied agile and dynamic motor skills [21] and locomotion control over challenging terrain [23] in legged robots, and achieved effective transfer from simulation training to real environments. In 2020, in-hand manipulation skills learned with RL demonstrated a high level of dexterity on a physical five-fingered hand [24]. Reinforcement learning has shown great superiority in solving nonlinear control problems, and has generated excitement in the robotics community. Moreover, the notion of reinforcement learning has similarities with animal learning progress, inspiring research on bionic robots. In the field of underwater robots, bionic robots are mainly applied in resource exploration, environmental monitoring, search and rescue, and other tasks that require motion control, sensor fusion, planning, and decision-making abilities, all of which can be further addressed by reinforcement learning. Due to the difficulties in modeling the underactuated bionic robot and the complex underwater environment, reinforcement learning is advantageous as it does not depend on the precise model of the robot. The innovation of RL techniques brings new opportunities for the research and development of bionic underwater robots.

Faced with different application scenarios, reinforcement learning algorithms have been extensively studied in terms of model-based methods, e.g., value iteration [25], policy iteration [26], and generalized policy iteration [27]; model-free methods, e.g., Monte Carlo methods [28] and temporal difference methods [29]; on-policy methods, e.g., TD and Sarsa [25]; off-policy methods, e.g., Q-learning [30] and Double Q-learning [31]; value function approximation methods, e.g., Fitted Q Iteration [32], LSMC [33], and LSTD [34]; policy function approximation methods, e.g., REINFORCE [35]; and so on. In addition, updates and improvements in deep reinforcement learning algorithms (DRL) [36] provide important support for establishing a complete intelligent control framework for bionic underwater robots.

The application of reinforcement learning to bionic robots has become a research hotspot, with the goal of enhancing autonomy and motion performance. In recent years, there has been significant progress in the cross-field of bionic underwater robots and reinforcement learning, with a particularly significant increase in related research since 2019. However, the research focus varies among the published work, and there are still certain barriers that researchers in the field of bionic underwater robots face when studying reinforcement learning methods. Therefore, taking into account the latest research progress, it is necessary to summarize the recent achievements of reinforcement-learning-based methods in the field of bionic underwater robots, and to indicate the challenges and future directions of reinforcement learning in this field.

This paper focuses on the application of RL-based methods in the field of bionic underwater robots, including the development of reinforcement learning algorithms, typical task scenarios of bionic fish, implementation methods for different bionic robot task scenarios, and deployment and training strategies on bionic underwater robot platforms. The remainder of this paper is organized as follows. In Section 2, the classification of typical reinforcement learning algorithms is introduced, and the latest reinforcement learning algorithms are listed. From the perspective of bionic underwater robots, Section 3 introduces the basic structures and task scenarios of bionic underwater robot platforms. In Section 4, reinforcement-learning-based methods for different task scenarios of bionic underwater robots are discussed specifically. Considering the distinctive characteristics of underwater environments and bionic robots, Section 5 summarizes the training and deployment strategies of reinforcement learning for bionic underwater robots. Finally, based on the latest research progress, Section 6 discusses the challenges and future directions of reinforcement learning in the field of bionic underwater robots.

## 2. Overview of Reinforcement Learning

In this section, we first introduce the basic concepts and principles of reinforcement learning, and then discuss some typical classifications of reinforcement learning algorithms from different perspectives, such as whether they are based on models, learning objectives, policy update methods, or approximator function types. We also specifically discuss reverse reinforcement learning and imitative learning when applied to complex practical tasks for which it is difficult to obtain reward functions. In addition, we summarize some advanced reinforcement learning algorithms that have demonstrated efficient learning frameworks and excellent training effects. Finally, we compare reinforcement learning with other learning-based methods and discuss its advantages when applied to robots.

### 2.1. Statement of Reinforcement Learning

The theory of reinforcement learning is inspired by natural animal behaviors. Through performing actions and interacting with the environment, organisms can transition from their current state to the next state, and receive feedback in the form of rewards. Organisms are capable of evaluating the quality of rewards, with positive rewards leading to the same action in similar situations, while negative rewards prompt exploration of alternative actions. This learning behavior observed in nature forms the theoretical basis for reinforcement learning, whereby better policies are learned through interactions with the environment.

### 2.2. Taxonomy of the Reinforcement Learning Algorithm

#### 2.2.1. Model-Based and Model-Free Algorithms

Reinforcement learning can be divided into model-based and model-free methods based on whether they rely on models. It is worth noting that both of these two methods obtain trajectory information by interacting with the environment. Model-based reinforcement learning methods use a complete state transition model and reward function to iteratively compute or solve the optimal policy; the most typical method is dynamic programming, which includes value iteration and policy iteration. When a complete model of the environment is known, dynamic programming can be used to iteratively find the optimal policy or value function; however, this approach may encounter issues such as a large state space and high computational complexity. Therefore, obtaining the optimal policy based on trajectory experience is necessary. On the other hand, model-free reinforcement learning methods can directly utilize the information obtained through interactions with the environment to continuously improve their policies without the need for modeling; typical algorithms include Monte Carlo (MC) and temporal difference methods (TD) [25]. These methods are more versatile and generalizable.

#### 2.2.2. Algorithms for Prediction Tasks and Control Tasks

Based on their learning objectives, reinforcement learning can be divided into prediction problems and control problems. For prediction problems, the value function of states is learned through value iteration, and the optimal policy is obtained by selecting the action set that maximizes the value for each state. In other words, at each state, the agent selects the action set with the highest value to obtain the optimal policy. For control problems, the goal is to obtain the optimal policy or value function in a Markov process, that is, an approximately optimal policy. The essence of reinforcement learning methods for control problems is the combination of prediction methods and policy iteration, where the prediction methods replace the policy evaluation step in policy iteration. Control problems are solved by estimating state values from trajectories and improving policies based on the estimated values to obtain the optimal policy. Typical algorithms include generalized policy iteration (GPI), Monte Carlo control, sequential differential control (Sarsa), and discrete strategy sequential differential control (Q-learning) [25,30].

#### 2.2.3. On-Policy and Off-Policy Algorithms

According to whether the policy updates and trajectory generation come from the same policy, reinforcement learning can be divided into on-policy learning and off-policy learning. On-policy learning essentially updates the policy π based on trajectory samples generated by the same policy π, which can guarantee the accuracy of the policy results. Off-policy learning, on the other hand, updates another policy μ based on trajectory samples generated by policy π, combining two different policies to make use of more unrelated data and improve training efficiency. For example, by pooling trajectory data produced in parallel by multiple robots with policies, $\pi_{1}$, $\pi_{2}$, etc., asynchronous policy updates can be achieved for the same task [37]. Common off-policy methods include Q-learning [30], Q(λ), and Double Q-learning [31].

#### 2.2.4. Approximator-Based Reinforcement Learning Algorithms

The methods described above have a basic premise that state space and action space are discrete, and the dimensions of these are not large. However, when faced with tasks with a large dimension of state space or a continuous state space, it can lead to problems such as excessive computational resource requirements and long computational time. Therefore, a function approximator is introduced to solve large-scale reinforcement learning problems. The value function is represented using function approximation, and then the reinforcement learning framework is constructed through strategy iteration and value iteration. By leveraging function approximation methods, agents can make predictions about unobserved states and take appropriate actions to maximize rewards, thereby improving their decision making in partially observable environments. According to the different approximation targets, these can be divided into value function approximators, strategy function approximators, and actor–critic algorithms [35].

Deep reinforcement learning (DRL) [36,38] utilizes artificial neural networks as function approximators, and leverages the advantages of deep neural networks in feature extraction to extract key features required for decision making in reinforcement learning, thereby addressing Markov decision making problems with complex high-dimensional inputs. DRL is currently a hot topic in reinforcement learning research. According to the different types of approximation functions, RL can be divided into value approximator-based algorithms, such as DQN, Double DQN, and Dueling DQN [25,36,39,40], and strategy approximator-based deep reinforcement learning, such as A3C [41], TRPO [42], PPO [43], DPG [44], and deep deterministic policy gradient (DDPG) [45]. It is worth noting that deep learning and reinforcement learning have different requirements for samples. In deep learning, it is assumed that the samples satisfy the independent and identically distributed condition, while reinforcement learning deals with Markovian temporal problems, where each sample $\left\{s_{i},a_{i},s_{i+1},r_{i}\right\}$ at time $t_{i}$ and $t_{i+1}$ has strong temporal correlation. Simply combining deep learning and reinforcement learning can have a certain impact on the convergence and stability of training. Therefore, deep reinforcement learning methods need to introduce techniques, such as experience replay and target networks, to reduce the temporal correlation between trajectory data [36,46], and thus achieve the integration of deep learning and reinforcement learning methods.

#### 2.2.5. Inverse Reinforcement Learning and Imitative Learning

While general reinforcement learning requires a known reward function, designing rewards for complex tasks can be challenging in real-world scenarios, hindering the direct application of reinforcement learning methods. Instead, expert data that do not rely on rewards are often easier to obtain in actual scenarios involving complex tasks. To address this challenge, this section introduces inverse reinforcement learning and imitation learning, which can avoid the need for manual reward setting and are worth discussing.

Reinforcement learning can be classified into forward reinforcement learning and inverse reinforcement learning based on whether the reward R is known. Forward reinforcement learning usually relies on a manually designed reward function, but there may be biases between the reward function and the optimal policy. Moreover, the reward function is often difficult to determine for complex tasks. In contrast, inverse reinforcement learning learns the implicit reward (i.e., the underlying objective function) from expert data samples, and then uses these rewards to train the reinforcement learning policy, thereby avoiding the limitations of manually designed reward functions [47,48,49]. Typical inverse reinforcement learning methods include maximum marginal inverse reinforcement learning, maximum entropy inverse reinforcement learning, and generative adversarial imitation learning [50,51,52].

Considering the limitations of reward function design, imitation learning is a technique that does not rely on environment reward. It utilizes supervised learning to directly train policies from observed data, thereby avoiding the reliance on models and environments. Specifically, in the presence of expert data, imitation learning obtains the desired agent policy through supervised learning, making the state–action trajectory distribution under this policy match the state–action trajectory distribution of expert data as closely as possible. The key techniques of imitation learning comprise of behavioral cloning, data augmentation, and dataset aggregation (DAGGER) [47,49,53].

### 2.3. Advanced Version of Reinforcement Learning Algorithms

With continued investment and research, reinforcement learning algorithms have achieved continuous iterative updates. In 2017, Haarnoja et al. proposed a soft Q-learning algorithm that represents strategies in the form of the value function softmax, enhancing the expressiveness of the strategy and ensuring complete exploration of the state-action space [54]. In 2016, Tamar et al. proposed the value iteration network (VIN), which considers the relationship between convolutional neural networks (CNNs) and value iteration, enabling the learned policy to focus more on long-term planning instead of just memorizing the one-step correspondence between states and actions in the short term [55]. VIN provides a solution to long-term decision making problems. In 2017, Van Seijen et al. proposed the hybrid reward architecture (HRA) method, which decomposes the reward of the environment and uses different action value functions to estimate the reward of different parts [56]. By decomposing the complex problem into several sub-problems, it addresses the issue of the total reward function being overly complex for learning. In 2017, Andrychowicz et al. proposed the hindsight experience replay (HER) algorithm, which addresses the challenge of exploration failures in scenarios with sparse rewards. The algorithm achieves this by adding failed explorations into an experience pool and then leveraging them to improve subsequent explorations [57].

The typical reinforcement learning methods are summarized in Table 1, providing specific category information for reference. In addition, Raffin et al. proposed stable-baselines3 [58], which established a well-encapsulated reinforcement learning algorithm repository and provided engineering significance for research based on RL methods.

### 2.4. Advantages of Reinforcement Learning

In the broader context of learning strategies, it is necessary to compare reinforcement learning with other learning methods, such as evolutionary algorithms and neural networks, to clarify the characteristics of reinforcement learning methods. Evolutionary algorithms (EAs) are a class of optimization algorithms inspired by the principles of biological evolution in nature. The basic idea is to start from a group of initial solutions generated randomly, which is referred to as the initial population, and gradually refine the solutions in the population through an iterative process. Common evolutionary algorithms include genetic algorithms (GAs), particle swarm optimization (PSO), differential evolution (DE), and others. Compared with reinforcement learning algorithms, evolutionary algorithms focus more on solving optimization problems, and are less suitable for sequential decision making problems. Additionally, the computational complexity and solution process of evolutionary algorithms make it difficult to achieve online decision making. The neural network algorithm is inspired by the biological nervous system, and learns to represent and process information by adjusting the connection weights between neurons. Neural network methods can handle highly nonlinear problems, but to some extent, they rely on supervised learning and require high computational resources, making them less suitable than reinforcement learning for online sequential decision making problems. Therefore, combining the two methods into deep reinforcement learning provides an effective approach for solving complex problems.

Overall, although there are still some limitations in terms of computational complexity, sample efficiency, and transferability, reinforcement learning algorithms have the following advantages for bionic underwater robots. First, reinforcement learning has online learning and real-time decision making capabilities, which are advantageous in robot control and policy adjustment. Second, deep reinforcement learning focuses more on the interaction between robot states, actions, and rewards, making it more advantageous for sequential decision making problems in robot control and planning tasks. Third, reinforcement learning methods do not rely on explicit labels, avoiding the problem of high cost in obtaining data during robot training. Fourth, deep reinforcement learning combines the advantages of neural networks and reinforcement learning, which is beneficial for solving high-dimensional and complex decision making problems, potentially significantly improving robot capabilities. Finally, reinforcement learning is robust to noise and uncertain environments, and has the potential to adapt to changes in complex environments.

## 3. Task Spaces of Bionic Underwater Robots

Before discussing the application of reinforcement learning algorithms in the field of bionic underwater robots, it is essential to understand the structure and task spaces of bionic underwater robots. This section first introduces the basic structure of bionic underwater robots, and proposes several task spaces according to the control–perception–decision framework. The learning tasks presented in this section face complex underwater environments and underactuated bionic robot scenarios. Therefore, the advantages and challenges of reinforcement learning algorithms in the field of bionic underwater robots are discussed at the end of this section.

### 3.1. Common Structure of Bionic Underwater Robots

**Figure 1:**
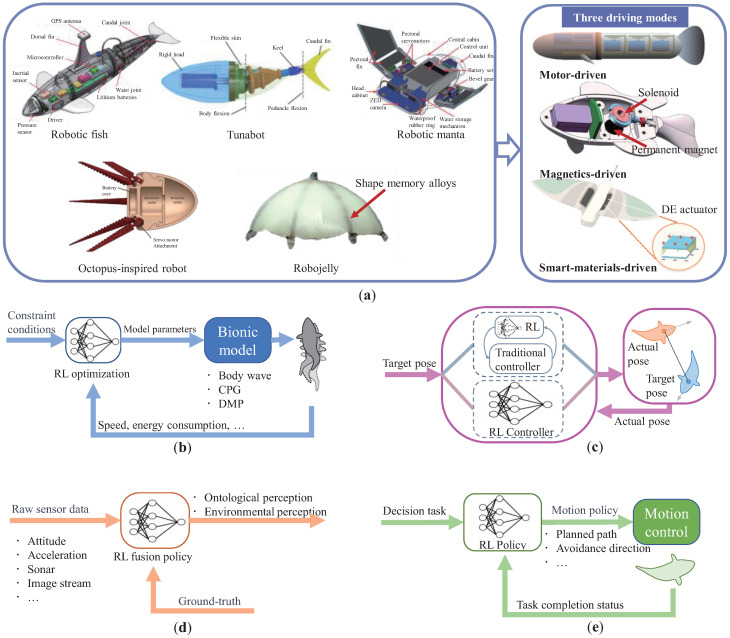
Common structure and task spaces of bionic underwater robots. (**a**) Classic structures of various bionic underwater robots and three driving methods. (**b**) Bionic action task. (**c**) Motion control task. (**d**) Perception fusion task. (**e**) Decision making task.

Bionic underwater robots mainly include flapping-inspired fish-like or dolphin-like robots [7,59,60]), jet-propelled bionic robots [61,62], and so on. The classic structures of bionic underwater robots are shown in Figure 1a. These robots are typically driven by electric motors, magnets, and soft materials to control the linkage of multiple components to achieve basic bionic actions [63]. The rhythmic motion achieved through the coordination of movable structures usually needs to approximate the motion of the biomimetic object as closely as possible in order to achieve similar high-efficiency motion performance. This fundamentally determines the mobility of bionic robots. The motion of bionic underwater robots is usually related to central pattern generators (CPGs) [64], and the simplified parametric model enhances the controlling ability of the bionic structure.

On the basis of achieving bionic actions, the bionic underwater robot swims in the water with the desired posture through the coordination of their functional structures, achieving high motion control performance. Underwater attitude adjustment mostly depends on the rudder-like functional structure, such as fin limb [65] and the center of gravity/buoyancy adjustment mechanism [66,67], and attitude control is the foundation for bionic underwater robots to complete complex tasks. Unlike mobile robots, underwater robots typically face three-dimensional motion scenes, while bionic robots typically have underactuated characteristics and exhibit periodic fluctuations in motion.

Bionic underwater robots usually combine five types of sensors, including motion positioning sensors (inertial measurement unit, depth sensor, Doppler velocimeter, etc.), environmental sensors (fish eye camera, sonar, lateral line system, etc.), communication sensors (radio frequency communication, sonar communication, etc.), power measurement sensors (power meter), and embedded auxiliary sensors (infrared sensor, reset sensor, force feedback device, water leak detector, etc.). Usually, bionic underwater robots realize the fusion perception of multi-channel data through core processors. Among them, the first two types of perceptual data are closely related to underwater missions.

On the basis of basic motion and perceptual data, bionic underwater robots can carry high-level algorithms, such as path planning, local obstacle avoidance, and target tracking, to improve the autonomous decision-making ability of the robot and complete difficult tasks. For example, bionic remoras hitchhike by attaching themselves to larger host fish [68], and bionic soft fish perform underwater search tasks [69]. In addition, in the bionic underwater robot swarm, decision planning algorithms are also required for formation control and pursuit and hunting tasks.

### 3.2. Task Spaces

The structure of bionic underwater robots determines the uniqueness of their task. Based on the structure of bionic underwater robots, task spaces are naturally divided into the bionic action control task, motion control task, fusion perception task, and decision making task. This section details the specific content of relevant research from the perspective of these four tasks.

#### 3.2.1. Bionic Action Control Task

The bionic action control task is closely related to biological inspiration. Previous research has revealed that basic animal behaviors, such as breathing, running, and so on, are likely to originate from spontaneous rhythmic signals of central pattern generators [64]. The central pattern generator (CPG) was then designed as a mathematical model, such as Hopf CPGs, Kuramoto CPGs, and so on, whose output is determined by a small number of model parameters, resulting in bionic action control. Most of the existing bionic action controls of bionic underwater robots are based on CPG, especially in multi-joint robots such as bionic snakes [70]. Dynamic motion primitives (DMPs) [71] is another bionic model similar to CPG, which can output various wave signals through supervised settings. In addition, for bionic robotic fish, the fish body wave model (FBW) [72] can also be used as a feasible model for studying bionic tail flapping motion.

Similar to the CPG model, both DMP and FBW are characterized by fewer model parameters, so the bionic action control task is often equivalent to the optimization of model parameters. The classical parameter optimization methods used for bionic action control models include artificial empirical optimization, particle swarm optimization [73], genetic algorithms [74], etc. These methods are generally suitable for solving fixed bionic model parameters, with better performance offline. It is difficult to adapt to changing environments, and online optimization has high computational costs.

Reinforcement learning can solve most optimization problems in specific models, and the optimization objectives are related to the rewards and punishments of RL. Therefore, reinforcement learning has a natural degree of adaptability to the parameter optimization problem of bionic action control. The framework for solving the bionic action control problem of the bionic underwater robot based on reinforcement learning is shown in Figure 1b.

#### 3.2.2. Motion Control Task

**Figure 2:**
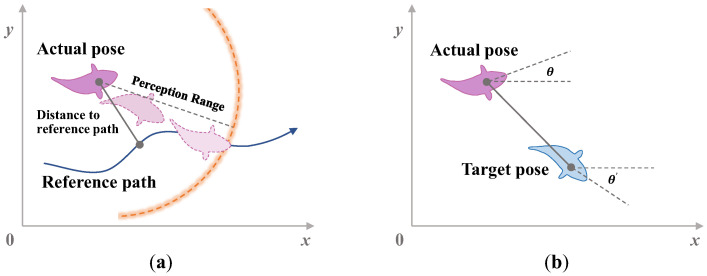
Motion control of a bionic underwater robot. (**a**) Path tracking. (**b**) Attitude control.

Motion control is an important prerequisite for autonomous operation of bionic underwater robots. The motion control task mainly focuses on the pose control and path tracking of bionic underwater robots, as shown in Figure 2. Under the constraints of different speeds, heading angle and pitch angle control jointly determine the three-dimensional motion of the bionic underwater robot. Due to the complex underwater environment and numerous unknown disturbances, the stable attitude control of bionic underwater robots is often focused on. Generally, the bionic propulsion has strong control coupling, and its control has certain difficulties.

In the field of bionic underwater robots, proportional–integral–derivative (PID), sliding mode control (SMC), fuzzy control, etc., are widely used in the motion control task [13,75,76]. Adaptive control and autodisturbance rejection control are also widely studied for the strong disturbance problem in the underwater environment [77,78,79]. Traditional control methods are influenced by the structure and parameters of the controller, resulting in mixed control effects. In recent years, model predictive control (MPC) has been used in nonlinear bionic robot motion control tasks, such as position control and tracking control, using the idea of open-loop optimal control. At the same time, intelligent control methods based on reinforcement learning have been tried by pioneers [80,81,82], with two main frameworks (shown in Figure 1c). Firstly, the reinforcement learning algorithm framework is integrated with model predictive control methods or other traditional control methods to obtain adaptive updates of the controller through the concept of interactive learning. Secondly, the deep reinforcement learning algorithm framework directly implements end-to-end control from perceptual information to control signals.

#### 3.2.3. Perception Fusion Task

The perception of the bionic underwater robot faces two problems. Firstly, the underwater environment has significant constraints on electromagnetic waves, light waves, etc., which greatly limits the perception range of GPS and visual systems. Secondly, bionic robots typically move periodically, causing periodic disturbances to sensory data. The task of perceptual fusion is to assess the state of a robot system or environment as accurately as possible based on data from various sensors, as shown in Figure 1d. Typically, calibrated sensor data are fused and adjusted through manually set rules for use in control or decision making tasks. The perceptual fusion task is typically considered as a subtask within the control or decision making algorithm.

#### 3.2.4. Planning and Decision Making Task

The decision making task of robots is closely related to the autonomous operation of robots. The decision making task can be subdivided into navigation, planning, search, tracking, scheduling, and so on. For the planning task of bionic underwater robots, the flapping range of the robot needs to be specially considered, especially in obstacle avoidance algorithms. For autonomous bionic underwater robots, the existing path planning and obstacle avoidance algorithms are relatively mature [83], but there is still a lack of a complete intelligent algorithm framework that integrates multiple decision making problems. The original intention of the deep reinforcement learning method is to deal with the decision making problems of agents. The RL algorithm has been widely used in unmanned aerial vehicles, autonomous vehicles, mobile robots, and other fields [84,85,86], which has reference value for the intelligent decision making of bionic underwater robots. The decision making and planning task framework of bionic underwater robots is summarized in Figure 1e.

### 3.3. Advantages and Challenges of RL-Based Methods

In recent years, reinforcement learning algorithms have been deeply researched, leading to new approaches to address tasks related to bionic underwater robots. Compared to existing methods, reinforcement learning methods have three main advantages in addressing the various task spaces mentioned above. First, the trial-and-error approach of reinforcement learning can stimulate the motion potential of bionic robots, enabling them to learn bionic actions that are difficult to achieve through manual intervention [87] and improve their motion performance [88]. Reinforcement learning methods support the autonomous and efficient “learning” of stable underwater swimming methods by robots. Secondly, conventional methods often rely on robot models, and have limited adaptability to complex environments. However, reinforcement learning methods rely less on models, and are expected to adapt to different underwater environments through perception. The adaptability of reinforcement learning algorithms to different environments and their low model dependency has greatly inspired researchers’ confidence in improving the performance of bionic underwater robots. Thirdly, reinforcement learning algorithms can be optimized online, and can achieve multiple tasks simultaneously by expanding the state vector and the action vector. Based on a complete reinforcement learning algorithm framework, bionic underwater robots are expected to achieve the “intelligence” of autonomy.

It is clear that reinforcement learning can cope with the task of bionic underwater robots; however, the specific implementation of the algorithm still faces four difficulties. First, training difficulties from simulations to the real world (sim-to-real) are conspicuous, with large underwater environment disturbances, and there is a lack of effective deployment methods for real environment training. While reinforcement learning based on training in simulation environments is challenging, ensuring the migration performance from simulations to the real world is even more difficult. Secondly, the accuracy of dynamic models of underwater robots is difficult, and even impossible, to achieve. Even model-free reinforcement learning methods require verification or pre-training in a simulation environment, and the establishment of the simulation environment cannot avoid the dependence of the dynamic model and the interaction with the underwater environment. Thirdly, the control stability of RL algorithms cannot be fully verified, which may generate harmful control instructions for the robot. Moreover, the communication efficiency in the underwater environment is low, and the information feedback of the robot during three-dimensional motion is inconsistent, and thus, the security of reinforcement learning algorithms running on robots is difficult to ensure. Fourthly, the rewards for some tasks are sparse, and RL algorithms converge slowly, making it difficult to achieve the desired results.

## 4. RL-Based Methods in Task Spaces of Bionic Underwater Robots

This section surveys the RL-based research for various bionic underwater robot tasks as fully as possible, although there may still be omissions. Table 2 summarizes the research motivations, RL algorithms, and performances reported by the related literature. From Table 2, most of the existing RL-based work is related to bionic action tasks, motion control tasks, and planning and decision making tasks, while RL-based perceptual fusion methods are less studied. Based on the current research, perceptual fusion tasks are mostly solved through other traditional methods, and are used as sub-processes of the other three tasks. Therefore, the discussion of the existing work in this section will no longer be conducted from the perspective of perceptual fusion tasks. In addition, the tasks of bionic underwater robots are hierarchical from top to bottom, and the design of upper-level tasks is usually related to the output of lower-level tasks. There are also some tasks that cross task levels in the researched literature. Therefore, this section mainly introduces three categories: bionic action control tasks, motion control tasks, and decision making tasks. Research involving cross-tasks will be separately focused on in related discussions.

### 4.1. RL for Bionic Action Control Tasks

Control tasks based on RL and bionic action models (CPG, DMP, FBW) usually aim at obtaining a better swimming gait. Swimming speed, mobility, energy consumption, etc., can be used to evaluate swimming gait. Based on the CPG model and RL methods, the robotic fish μBot learned a swimming gait with backpropagating traveling waves, with the goal of maximizing its swimming speed [89]. Based on the FBW model, RL method SAC provided an effective approach for obtaining subcarangiform body wave parameters for a five-jointed fish-like robot, and both the cost of transport and velocity performance were optimized [90], in which the parameters of two optimized groups were compared, verifying that the optimal efficiency and optimal speed cannot be achieved simultaneously. Based on the DMP model and trust region policy optimization (TRPO) method, the robotic tadpole achieved an effective propulsion gait, with expected thrust and stable heading attitude as high rewards [91], which generated a target point strategy for the DMP model through navigation learning, allowing the robot to swim along a number of randomly generated paths. No matter which kind of bionic motion model, an appropriate reinforcement learning algorithm design can achieve gait optimization for bionic underwater robots.

The most highly regarded evaluation criterion for bionic locomotion is undoubtedly energy efficiency optimization. To optimize the energy efficiency of a bionic robotic fish, the method proposed in [92] is based on the flow field sensor in the fish tail (which provides low-dimensional force feedback signals), and relies solely on proprioception to perceive the robot’s undulation state, in which energy consumption was effectively reduced based on improved CPG parameters. Similarly, aiming to optimize energy efficiency, ref. [93] constructed a two-stage reward function based on an adversarial model that includes two competing gliding robotic fish. This method saved approximately 4.88% of energy and about 19.45% of traveling time. Based on a two-segment linear drive structure, ref. [97] designed a robotic eel and used the SAC algorithm to achieve swimming control based on the geometric relationship of the mechanism. In this method, in addition to energy consumption, the straight swimming speed and swimming deviation are also included in the reward function. Ultimately, the control strategy with maximum speed and suboptimal energy efficiency was chosen. These two performance optimization cases indicate that, for bionic underwater robots, performance optimization does not rely solely on the optimization of a single performance factor. Instead, solutions that consider multiple performance factors are often preferred. Reinforcement learning methods also have the capability of handling optimization problems that take multiple performance factors into account.

The complex structure of multi-joint robots makes it difficult to manually tune the parameters of their bionic action models. In 2006, Liu et al. applied RL algorithms based on AC networks to the multi-joint robotic fish MT1 Profile, and tuned six parameters of the bionic action control model, achieving effective turning speed [94]. In 2022, two works [90,123] applied RL algorithms to tune the model parameters of fish body wave and CPG in five-jointed robotic fish and four-jointed robotic dogfish, respectively. Reinforcement-learning-based methods undoubtedly provide effective solutions for the parameter tuning problem of multi-joint robots.

When CPG is used as the bionic action model, the smooth transition of the robot in different swimming modes is related to the convergence speed of CPG. High convergence speed is prone to oscillation, while low convergence speed is not conducive to modal transition. Optimizing the convergence speed of CPG through reinforcement learning algorithms is a developing research direction. In 2022, based on the improved CPG with a chain coupling of 16 oscillators with bidirectional perturbation, RL algorithms were adopted to achieve natural modal switching of elongated undulating fin propulsion [124]. Specifically, Q-learning uses oscillation error as rewards and punishments to search for optimal convergence speed, achieving performance improvement in CPG [124]. On the other hand [88], the optimization of DDPG not only improved the convergence speed of the CPG network by about 2.2%, but also achieved higher amplitude precision compared to the DQN algorithm (about 1.6%), leading to high efficiency in controlling the swimming gait of the robotic fish. In addition, from Table 2, action control based on the CPG model has an advantage in terms of quantity. To expand the application value of CPG in reinforcement learning, improved CPG algorithms have also been proposed to meet control requirements and reduce the risk of abrupt changes in parameters, such as normalized CPG [125], modified CPG network in bidirectional perturbation [124], and modified CPG with reduced input parameters [98]. These novel CPG models are more suitable for reinforcement learning environments, providing a solid foundation for subsequent RL-based bionic motion optimization methods, and are of high reference value.

For soft-bodied robots that exhibit viscoelasticity and extensive deformation, appropriate actions may not always be manifested, even if the body dynamics are given. This is challenging to control, and RL methods have received attention. Ref. [126] constructed soft-bodied animals with bionic actuators, and used RL to imitate the movement of soft-bodied animals as much as possible. Ref. [127] leveraged the scalability of RL to enable soft robots to explore a variety of behaviors automatically. The motion control research for soft robots using RL is discussed in Section 4.2.

Additionally, due to the exploratory nature of RL, some studies on bionic action control have led to a deeper understanding of biological mechanisms. For example, Deng et al. [89] systematically explored the potential relationship between body morphology, swimming gait, and swimming performance through RL, and confirmed that the shape of the caudal fin has a certain influence on gait and swimming speed. Zhang et al. [95] found that, after numerous trials and errors, RL training for flapping motion always converges to patterns that are similar to harmonic motions, proving that harmonic motion with appropriate amplitude and frequency is always an optimal choice for efficient underwater propulsion. Li et al. [92] proposed that even with a damaged lateral line system, relying solely on the flow field perception of the fish tail is sufficient to optimize energy efficiency. The additional knowledge obtained from RL-based experiments is exciting. Therefore, in suitable task scenarios, reinforcement learning methods are recommended to explore deeper inferences.

### 4.2. RL for Motion Control Tasks

Motion control can be further classified into attitude control and position control. Attitude control is based on either an inertial measurement unit (IMU) or a lateral line system (LLS). IMU-based attitude control focuses on controlling and stabilizing the attitude angles of the roll, pitch, and yaw of the robot, while LLS-based attitude control is related to flow field perception, such as flow velocity and angle of attack [17]. RL-based attitude control is one of the basic research areas of motion control. In [99], bionic RoboDact is controlled to maintain a target yaw angle and speed from a random attitude and non-motion state by designing relevant rewards, and training and validating the SAC controller in simulation. In [100], an effective solution for the attitude control of the jellyfish-like robot relies on both a 3D barycenter adjustment mechanism and a Q-learning-based attitude control method, whose reward is related to the target and the current attitude; however, freely adjusting the three-axis attitude has not yet been achieved. Unlike the previous two studies focusing on the overall attitude of bionic robots, the study in [101] focuses on the posture of each soft arm of an octopus-inspired soft robot. Based on a set posture error thresholds, precise attitude control of the soft arm is achieved through the DQN method, and the bipedal walking of the octopus-inspired soft robot is realized by coordinating the two precise attitude-controlled soft arms. Another posture control example focuses on buoyancy-driven underwater gliders, combining a natural actor–critic (NAC) algorithm with an active disturbance rejection control (ADRC) [102]. The parameters of ADRC are adjusted by the NAC method, which compensates for ocean current disturbances and achieves high precision and highly adaptive attitude control ability.

Position control can be divided into planar position control and depth control. Specifically, the depth control of bionic underwater robots is often related to pitch attitude. For bionic underwater robots, depth control influences underwater three-dimensional (3D) motion performance, and is one of the research focuses. In order to avoid relying on the mathematical model of the bionic manta, ref. [103] trained a controller based on Q algorithm data and transplanted it into the robot prototype to conduct depth control. Another depth control scheme, the MPC-based DDPG control algorithm, is implemented in the bionic penguin platform [104]. This scheme builds a data-driven MPC depth control framework, and the reinforcement learning algorithm optimizes its approximation of the optimal control while ensuring control safety and stability, achieving significant control effects. Whether using end-to-end RL controllers or improved traditional controllers based on RL, existing research has verified the effectiveness of introducing RL in the pose control of bionic underwater robots. The inclusion of reinforcement learning has, to some extent, improved the accuracy of pose control and the ability to adapt to the environment.

Taking into account both attitude control and position control, 2D or 3D path tracking is a common motion control task for bionic underwater robots [98,105,106,107,108,109,110,111,128,129] that enables the motion capability of bionic robots in underwater environments. The authors of [111] deployed the DDQN algorithm to the path tracking control of a hybrid-driven robotic fish, and quantitatively compared the control performance of RL with PID and SMC. To our knowledge, this study is the first to adopt this technique. Meanwhile, the study by [105] verified three RL algorithms of PPO, A2C, and DQN in the motion control environment of a soft bionic Pangasius fish robot, and ultimately, the PPO agent performed better. Similarly, a cooperative structured control based on evolutionary strategy and DDPG is proposed for the 3D trajectory tracking control of bionic robotic fish, saving 23.97%, 22.13%, and 38.72% energy compared with SMC, ADRC, and PID, respectively. In addition, for stable path tracking control, ref. [112] designed a bionic robotic fish with a reaction wheel, and controlled the momentum wheel and tail flapping at different frequencies using multi-agent RL. For complex tasks that consider both position and attitude control, reinforcement learning exhibits strong performance, and can effectively address multi-objective control problems. In particular, motion control based on RL has been widely researched and validated in the field of soft robotics [81,82,105,107]. For instance, the multi-objective control problem of the soft robotic fish with a bundled SCP actuator, which includes heading control and path tracking, was solved by a DDPG controller and a linear–quadratic-regulator-based multi-objective reward mechanism [107]. Based on model-free SAC, the soft robot proposed in reference [81] learned to move in a straight line in disturbed water.

In certain tracking scenarios, desired speed tracking control has been focused [113]. Swimming speed, as one of the important motion performance indicators, is difficult to measure and usually indirectly obtained, with significant data noise. In [113], both DDPG controller and twin delayed DDPG (TD3) controller methods were conducted based on the swimming speed data from simulations or a global camera. In contrast, TD3 avoids becoming trapped in local optima and improves the control speed tracking accuracy. Earlier, in [82], a Q-learning-based speed control method was conducted on a three-link soft robotic fish actuated by antagonistic artificial muscles, which is expected to be generalizable to many other robot speed control problems, since it does not rely on accurate dynamic models.

For bionic underwater robot platforms equipped with vision systems, target tracking control has important research value. Based on stable visual information and target position, reinforcement learning algorithms take target position as the input, CPG parameters as the output, and achieve the continuous tracking of a selected target without the need for the robot’s dynamics knowledge [108]. Similar results were achieved in [106].

In the field of motion control for robotic fish, Xie’s team has worked on a DRL-based control method [17,18,98,114], demonstrating a complete and informative RL motion control framework. To address an unknown flow field, the study by [17] fuses data from LLS and IMU, and trains a data-driven simulation environment based on DDPG, holding a desired angle of attack. In addition, the studies [18,98] focus on path tracking and pose control, and train in both the surrogate environment and the CFD environment based on A2C, improving the efficiency of RL training and the precision of underwater control experiments. To balance position control and attitude control, ref. [114] only rewarded the robot when it reached the desired pose, and reduced the difficulty of training with imitation learning. The algorithm exhibited robustness in disturbed underwater experiments. The above three works have complete frameworks and implementation steps, providing guidance and foundation for researchers in the cross-disciplinary field of robotic fish and reinforcement learning.

In conclusion, RL research on bionic underwater robot motion control task can be divided into parameter optimization RL control methods and direct RL control methods. The former mainly relies on traditional control methods, with RL as a supplement to improve traditional control methods, such as ADRC-based NAC controller [102], MPC-based DDPG controller [104], and PID-based SAC controller [128]. The latter directly builds a control framework based on RL, i.e., end-to-end RL controllers, to train the motion control capabilities and improve control performance, such as [17]. Comparing the two control frameworks, the data flow logic of the former is determined by the structure of the set traditional control method, while the data flow logic of the latter is related to the designed deep network. Although it is not possible to conclude from the surveyed literature which one of the RL routes above is superior for motion control, it is certain that the participation of reinforcement learning has the potential to explore and improve control performance [109,111].

### 4.3. RL for Planning and Decision Making Tasks

Compared to bionic action control tasks and motion control tasks, decision making tasks for bionic underwater robots are more diverse, such as searching [69], obstacle avoidance [115], formation control [116,117], and other swarm strategies [118,119,120]. The majority of current research on RL-based decision making for bionic underwater robots is conducted in simulation environments. However, in the case of bionic underwater robots, the periodic envelope of bionic flapping, especially in obstacle avoidance problems, needs to be considered by RL-based decision making methods. In addition, the disturbance factors in the underwater environment must be considered as safety elements.

In decision making tasks, underwater searching has practical application prospects. For the task of searching the water tank boundary, model-based Q-learning was conducted with G9 robotic fish [69]. In [130], based on an intelligent visual servo Q-learning algorithm, the bionic soft robot for target searching was trained by a threshold reward system, which takes a certain degree of tolerance for target pointing errors. In addition, a three-stage real-world deep RL framework was proposed to achieve underwater autonomous exploration of robotic sharks [87], in which real-world training improves the adaptability to sensor noise and the real-world environment, achieving a safe and efficient underwater autonomous search event.

From the perspective of formation control tasks, studies have shown that RL methods can explore the energy-saving mechanism of machine fish formation in fluid environments [116], that is, efficient swimming is achieved through the eddies of adjacent regions, which is inspiring for the study of cluster energy efficiency. For decentralized circle formation control for fish-like robots, a new MARL method based on value decomposition networks (VDN) was proposed [16], and the cognitive consistency of multi-agents realized by parameter sharing and the centralized training mechanism with decentralized execution is an important factor in the effective formation of control methods. A dueling double DQN (D3QN)-based approach in the leader–follower topology was proposed for end-to-end formation control [117], and the blindness of agent exploration at the beginning of training was reduced through imitation learning. Similarly, ref. [120] discussed the leadership strategy of machine fish for real agents.

From the perspective of obstacle avoidance tasks, a one-step actor–critic-based obstacle avoidance algorithm for self-propelled fish was designed in [115], which controls the robot to avoid multiple obstacles. In addition, an interesting water polo ball heading strategy for robotic fish with hybrid fin propulsion was proposed [121], which decomposes the action and is implemented based on the SAC method. Robotic fish adversarial/cooperative problems, such as chase–escape games [119] and “2v2 games” [118], are also discussed. Reinforcement learning is highly suitable for solving decision making problems. However, relevant research on bionic underwater robots is still insufficient. A large number of decision making and planning tasks need to be further explored to enhance the individual intelligence of bionic robots.

## 5. Training and Deployment Methods of RL on Bionic Underwater Robots

In the training and deployment of bionic underwater robots, reinforcement-learning-based methods face two main challenges. First, the underwater environment is complex, making modeling difficult, and the transfer performance from simulation to real environments is uncertain. Second, the computational complexity of algorithms supported by resource-limited bionic robot systems is limited. Therefore, this section summarizes the training methods, deployment methods, and training techniques in the related literature, and discusses the computational complexity of reinforcement learning algorithms.

### 5.1. Training and Deployment Framework

Analyzing the specific implementation of RL methods in Table 2 from a statistical perspective, many studies have attempted to train intelligent agents in simulation environments and directly transfer them to underwater environments for validation. The effective transfer of RL algorithms from training to deployment is a major aspect of research focus.According to the surveyed works, effective training and deployment frameworks can be categorized into five types, as shown in Table 3.

There are relatively few studies based on real-world training deployment, as presented in Table 2, excluding those that were trained directly in a real-world environment without pre-training. The convergence speed and training accuracy are two objectives of RL training, as mentioned in [81]. The combination of pre-training and high-precision training can improve training speed in the early stages and training accuracy in the later stages. The aforementioned three training deployment frameworks all reflect this approach, and the actual algorithm performance also verifies its effectiveness.

**Figure 3:**
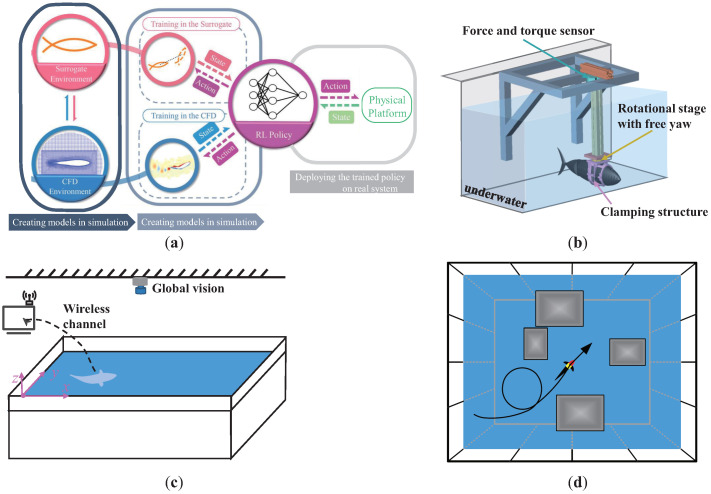
RL training and deployment environment for bionic underwater robots. (**a**) Self-switching simulator (Tri-S) system [18]. (**b**) Underwater semi-fixed training platform based on mechanical sensors. (**c**) Underwater deployment environment based on global vision. (**d**) Diagram of the real-world training environment [87].

Figure 3 presents several valuable training environments for bionic underwater robots. The self-switching simulator (Tri-S) system, shown in Figure 3a, is suitable for RL-based decision tasks that are difficult to complete in real environments; however, it relies on precise CFD models to ensure the performance in real environments. The semi-fixed underwater training platform with yaw freedom is based on force sensors, as shown in Figure 3b, which is suitable for RL-based bionic action control tasks. The reward feedback related to propulsion force, heading angle, and energy consumption can be obtained through the force sensor and onboard sensors. RL training based on this platform allows interaction with the real underwater environment, ensuring the deployment accuracy of the method. The underwater deployment environment based on global vision, shown in Figure 3c, is suitable for position control or path tracking control tasks, and the global vision system can calculate the robot’s pose, providing conditions for RL algorithm deployment. However, training on this platform still poses some difficulties, and the processing delay of visual signals may not meet the control frequency of high-swimming-speed underwater robots. If this environment is to be used for real-world training, sufficient and safe termination conditions are necessary. The obstacle environment based on real-world environments, shown in Figure 3d, is suitable for training underwater decision tasks, such as target searching and path planning, provided that the bionic underwater robot itself has complete perception feedback required for the task.

**Figure 4:**
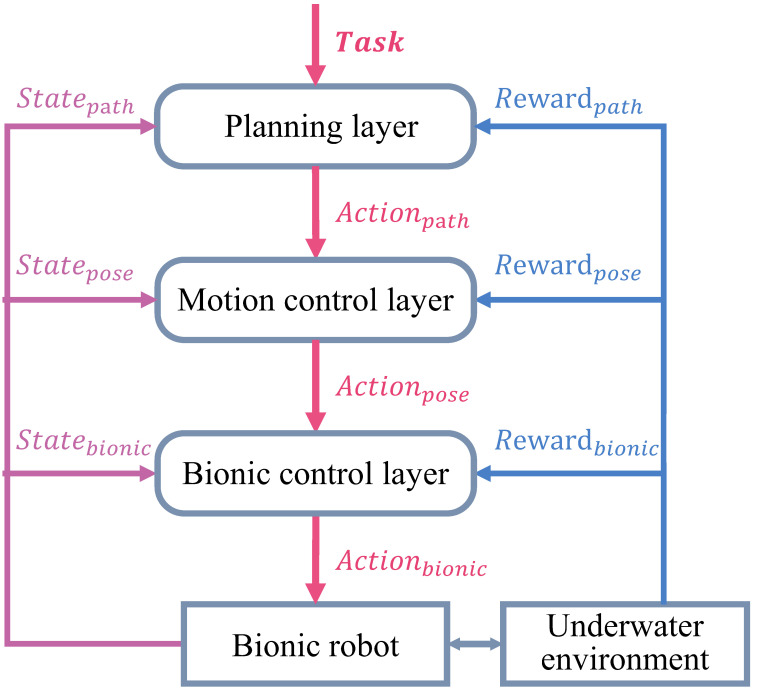
Hierarchical reinforcement learning framework.

Combined control and decision tasks for bionic underwater robots are complex. From the perspective of the existing RL algorithm, the hierarchical RL framework [132], as shown in Figure 4, is conducive to reducing the curse of dimensionality, improving the performance of each layer of algorithms in turn, and helping to build a complete, intelligent, bionic underwater robot system.

### 5.2. Training Techniques

Effective training techniques are beneficial for improving the performance of RL algorithms in bionic underwater robot tasks. For example, four strategies, i.e., prioritized experience replay, actor network indirect supervision training, target network updating with different periods, and expansion of exploration space by applying random noise, were applied in [106], respectively, to eliminate the correlation of training data, ensure the stability and speed of the convergence of the reinforcement learning AC network, update the critic network faster, and more accurately evaluate and improve the actor network’s generalization ability. Highly correlated data may lead to local convergence in RL [133]. One solution is to perform random sampling in the experience replay buffer, but this solution is only suitable for off-policy RL [133]. Another solution is multi-agent RL. With the increase in the number of agents, the computational complexity of multi-agent RL also increases.

To facilitate overcoming difficulties during the training process, more detailed techniques have been summarized as follows. To avoid the blindness of the agent improvement in the early stage of training, an imitation-based action selection strategy [117] and teaching initialization [87] are used in RL algorithm training. In order to prevent the RL algorithm from being trapped in the optimum and to accelerate the training, the go-explore strategy in [95] is used, which records the encountered states into an archive and replays them at the beginning of subsequent episodes. These two training techniques are relatively common in RL methods. However, when facing underwater environments and bionic platforms, more techniques are required.

Two training techniques were adopted in [93] to obtain a superior solution: the adversarial model and a two-stage reward function. The adversarial model calculates the reward at each step based on the performance parameters of two competing robots, while the two-stage reward strategy designs two different rewards for each step distance and each episode power consumption to balance the two optimization objectives. In addition, the hierarchical training method, including initial training and iterative training, was proposed in [112] to deal with the control coupling and frequency difference between two agents in multi-agent reinforcement learning methods. During training in [97], a random disturbance was added to the position, velocity, and angle of the joints of the initial robot in each episode, to increase the adaptability to the initial perturbation state. In addition, most bionic underwater robots have periodic motion, so real-time output of the reinforcement learning control algorithm is not necessary, and periodic control output is more suitable for the needs of bionic underwater robots. The learned policy’s action distribution via regression is fitted as mathematical functions in [134], so that the reinforcement learning control strategy can be fine-tuned after algorithm deployment. Moreover, centralized training with decentralized execution (CTDE) is a common training paradigm for swarm tasks [16].

The goal of [130] differs from other approaches aimed at improving control accuracy, as it employs a threshold reward system that demonstrates a certain degree of tolerance for underwater tracking errors. In underwater turbulent environments, precise motion to a specific location is difficult, and in underwater search tasks, small tracking errors can be tolerated. Therefore, the threshold reward system [130] not only helps to reduce the training difficulty reasonably, but also encourages the agent to approach the target with a natural intuition.

### 5.3. Computational Complexity

The computational complexity of RL algorithms usually needs to consider two aspects: the computational complexity during training and the computational complexity during deployment. The computational complexity during training is related to the specific RL method chosen, the training environment, and the algorithm convergence speed, which usually involves complex calculations and takes a long time. On the other hand, the computational complexity during deployment for some RL algorithms often only requires a few matrix operations to be completed [18]. Bionic underwater robot platforms usually have a small hull and limited computing power. Therefore, evaluating the computational complexity of RL algorithms in bionic underwater robots is crucial, as it determines whether they can be deployed in real bionic underwater robot systems, as well as the real-time performance of the algorithm after deployment.

In the surveyed works, the computational complexity and real-time deployment of RL algorithms in the field of bionic underwater robots are rarely discussed. In [17], based on the DDPG policy, the deployment computational complexity based on a five-layer deep neural network was carefully calculated, which is approximately at the order of $10^{3}$, and the running time based on a microcontroller (STM32F103) is about 10 ms, which can be directly deployed onto the robotic fish. In addition, ref. [18] pointed out the training period of the proposed algorithm. The whole learning process takes 16 days, where the first 350 episodes within the surrogate environment take only 50 min [18]. That is, the learning process for just 50 episodes with the CFD environment needs 16 days, which confirms the computational complexity during training. It is worth noting that its computational complexity is related to the CFD-based simulation training environment.

## 6. Challenges and Future Trends

Research on reinforcement learning methods for bionic underwater robots is currently still in its early stages, and faces numerous challenges posed by underwater environments and bionic systems. This section will discuss these challenges and future trends in two parts, hoping to provide some feasible directions for future development.

### 6.1. Challenges

#### 6.1.1. Inevitable Modeling

It should be noted that the meaning of “model” in “robot model” is different from that in “model-free” of reinforcement learning (RL). The former is usually built based on dynamic analysis, CFD models, or numerical simulation, and is used to construct simulation training environments. The latter is related to the state transition information of RL agents. Most of the RL training or pre-training of robots relies on simulation environments. Therefore, even if model-free RL methods are used to design controllers or decision-makers, it is still necessary to configure the simulation environment based on the model of the specific bionic underwater robot platform.

#### 6.1.2. Transferability to the Real-World Environment

The transferability of RL algorithms from simulations to the real-world underwater environment is generally uncertain. The simulation training environment for bionic underwater robots faces challenges in modeling underwater fluids and the robots themselves. Simulation training environments based on the dynamics model of bionic underwater robots have high training efficiencies, but often cannot maintain identical performances to those of simulations when deployed in the real-world, making it difficult to evaluate their sim-to-real performance. Numerically driven simulation training environments can further reduce the gap between simulations and reality [18], but require large amounts of physical data acquisition. CFD-based simulation training environments can improve the training accuracy of the simulation environment, but rely heavily on computing resources, and can be time-consuming [135,136]. However, training bionic underwater robots directly deployed in the real environment is not yet widely conducted, and lacks mature solutions. In summary, designing RL algorithms that ensure transferability remains a challenge.

#### 6.1.3. Sample Efficiency of Training

RL algorithms usually require a large number of interactions with the environment to learn the optimal policy. In underwater robots, data collection is expensive and time-consuming, making it a significant challenge to improve sample efficiency. In particular, training bionic underwater robots in real-world environments requires higher monitoring and time costs for robot–environment interactions. Therefore, building an efficient training/deployment framework remains a challenge for RL research on bionic underwater robots.

#### 6.1.4. Security of Deploying RL in Underwater Environments

For bionic underwater robots, the underwater environment has a cushioning effect on the impacts the robot’s body, which is advantageous for the robot itself. However, when applied to unknown environments, the robot may act in unconventional or dangerous ways during the RL-based learning process [96]. At the same time, the safety of the decision outputs by the RL agent cannot be traversally verified, and the RL-based bionic underwater robot control or decision task executor may guide the robot to the wrong area, resulting in the damage or loss of the robot. The security of algorithm deployment presents a critical challenge in the application of RL to bionic underwater robots.

#### 6.1.5. Robustness and Adaptability for Continuous Disturbances in Underwater Environments

Underwater environments typically have continuous, unstructured disturbances, which pose high demands on the performance of RL algorithms in unknown environments. Firstly, RL algorithms need to face the impact of factors such as water flow, water pressure, and limited visibility in underwater environments. Secondly, underwater environments have continuous interference and noise, making RL methods that can adapt to changing environments necessary. A Bayesian method for handling uncertainty [137] has been attempted for RL-based anti-disturbance control, but its computation is complex. Overall, the design of RL methods for continuous disturbances in underwater environments needs to consider two factors: on one hand, simulate the disturbances of underwater environments in the training environment, and on the other hand, improve the performance of the system in disturbance environments through reasonable reward settings. Maintaining the performance of RL in underwater environments with disturbances remains a challenging issue.

#### 6.1.6. Computational Complexity and Online Deployment

Autonomous bionic underwater robots are expected to achieve “intelligence” through reinforcement learning algorithms, and online optimization of agents after deployment helps the bionic robot achieve continuous improvement in its decision making ability in different environments. However, the computational resources of the bionic underwater robot platform are limited, and the complexity of the computations it can perform is limited. The time cost of training and the computational cost of algorithm deployment both determine whether an intelligent agent can be updated and optimized online. In addition, online updates affect the lifespan, durability, and properties of the robot, and the motion wear, malfunctions, and other issues of the robot’s mechanism may interrupt optimization.

### 6.2. Future Trends

#### 6.2.1. Multiple Bionic Motion Combination Control

The current bionic action control methods only learn periodic basic actions, while the swimming motion of real fish is often a combination of several basic actions [138]. We know that the efficient swimming of fish is related to their ability to conform to the flow field while flapping, and for bionic underwater robots, the combination of multiple swimming motions helps to achieve more precise bio-inspired propulsion, resulting in more efficient propulsion and higher swimming maneuverability. The optimization of bionic motion combinations is of great research significance for bionic underwater robots. Reinforcement learning methods are good at making decisions for combination actions, and under reasonable rewards and action configurations, bionic underwater robots are expected to achieve better propulsion performance.

#### 6.2.2. Applicable Training Schemes for Underwater Environments

Reinforcement learning provides the conditions for the intelligence of bionic underwater robots. However, bionic underwater robots are characterized by underactuation, and are required to navigate complex underwater environments, which poses challenges to ensuring the safety of intelligent agents operating in such environments. At present, the training framework for bionic underwater robots is not complete, especially in terms of performance in real underwater environments. Therefore, due to the particularity of bionic underwater robots, research and deployment of reinforcement learning algorithms tailored to local conditions still needs to be continuously advanced.

#### 6.2.3. Improving the Transferability and Generalization of RL

Adaptation and domain randomization are techniques in reinforcement learning used to improve algorithm generalization performance and achieve simulation-to-reality transfer. The adaptation technique aims to enable learning algorithms to transfer between different environments or tasks, including domain adaptation and policy adaptation. Domain randomization, on the other hand, randomly changes parameters in the simulation environment during the training process, such as physical properties and environmental conditions, to enable the learned policy to adapt to various possible changes. By training agents to face a large number of different situations, they are forced to learn more robust and general policies. By applying adaptation and domain randomization techniques, reinforcement learning algorithms can learn policies with stronger generalization ability in the simulation environment, leading to better performance in the real environment, which is an important area for future research.

#### 6.2.4. General Simulation Training Environment

Currently, reinforcement learning methods in the field of bionic underwater robots have made initial research progress. However, many of the proposed methods are difficult to compare directly, and the pros and cons of the algorithms are difficult to discern. In 2022, a novel RL training platform FishGym was designed, as reported in [139], based on a localized, two-way coupled fluid–structure interaction simulation model, and equipped with reinforcement learning components. Inspired by this, if bionic underwater robot platforms can be precisely established as Gym environments through digital twin technology, researchers in the field of reinforcement learning can become more specialized in obtaining higher performances of RL algorithms, laying a foundation for further research on RL intelligent algorithms.

#### 6.2.5. Performance Evaluation System of RL in the Field of Bionic Underwater Robots

According to statistical data, most RL-based control and decision making methods only verify their effectiveness and feasibility [99,140], and only a few compare RL with other traditional methods. On the one hand, the evaluation criteria for RL algorithms in the field of bionic underwater robots are unknown, and there is a lack of a baseline. On the other hand, the algorithms for different robot platforms are diverse and difficult to reproduce, so the recognized performance evaluation indicators in the field deserve further clarification.

#### 6.2.6. Complete RL-Based Algorithm Framework for Bionic Underwater Robots

For a complete task of an bionic underwater robot, task layering can effectively achieve the reduction in state space dimensions and solve the problem of dimensionality catastrophe [141]. Hierarchical reinforcement learning is committed to decomposing complex reinforcement learning problems into multiple sub-problems, and solving them separately to achieve better results than directly solving the entire problem. Hierarchical reinforcement learning and multi-agent reinforcement learning methods are suitable for building a complete algorithm framework for bionic underwater robots. In addition, the neural networks of animals are mostly spiking neural networks, and spiking neural networks [129] help to achieve the selection of 0/1 actions in the DRL framework, such as the selection between power-saving mode and full-power mode. Further in-depth research based on DRL is needed to develop a complete intelligent algorithm framework for bionic underwater intelligent robots.

#### 6.2.7. Individual Intelligence of Bionic Underwater Robots

Bionic underwater robots have important application values in water resources exploration, underwater searching, and other aspects. The improvement of the individual intelligence of bionic underwater robots is of great significance for their independent completion of complex underwater tasks. Reinforcement learning can provide online decision support and optimization suggestions for bionic underwater robots, and also has the ability to adapt to changing tasks and environments, which can assist bionic underwater robots in maintaining robustness and fault tolerance under uncertain factors such as communication interruption and hardware failure. Reinforcement learning has the potential to bring significant advances to the applications of bionic underwater robots, addressing the challenges faced by individual intelligence and providing new possibilities for the development of bionic underwater robot technology in the future.

#### 6.2.8. Multi-Agent Collaboration and Coordination

In many underwater tasks, a single robot may find it difficult to complete the task or have low efficiency, so multiple robots are needed to work together. The study of bionic underwater robot swarm is one of the hotspots in the field. In this case, it is crucial to design reinforcement learning algorithms that can achieve multi-agent collaboration and coordination. In multi-agent learning, shared or decentralized learning methods can help solve the coordination problem of multiple robots. At the same time, meta-learning, online learning, or transfer learning methods can make the multi-agent system more adaptable and have stronger learning ability. In the future, developing RL algorithms that can effectively handle multi-agent scenarios and promote cooperation is a promising research direction.

## 7. Conclusions

Reinforcement learning shares a similar concept with biological evolution, and has significant research value in the field of bionic underwater robots. Currently, reinforcement learning research has been widely applied to bionic action control, motion control, planning, and decision making of bionic underwater robots, which are exposed to complex underwater environments and are underactuated. Feasible algorithm designs have been proposed for specific bionic underwater robot platforms, and the available research on training and deployment frameworks is worth referring to. The intersection of bionic underwater robots and RL is still in its initial stages and requires further exploration.

In the future, the development of reinforcement learning in the bionic underwater robot community depends on mature training and deployment solutions, innovative high-performance RL algorithms, and well-known platforms or evaluation systems. In addition, the individual and collective intelligence of bionic underwater robots relies on the sequential decision making and cooperation coordination ability of reinforcement learning. The development in this field is promising.

## Figures and Tables

**Table 1:** Typical reinforcement learning algorithms.

Algorithms	Model-Based/Model-Free	Learning Task	On-Policy/Off-Policy
Policy iteration	Model-based	State value	—
Value iteration	Model-based	State value	—
Monte Carlo method (MC)	Model-free	State value	On-policy
Temporal difference method (TD)	Model-free	State value	On-policy
Sarsa	Model-free	Optimal value function	On-policy
Q-learning	Model-free	Optimal value function	Off-policy
Q(λ)	Model-free	Optimal value function	Off-policy
Double Q-learning	Model-free	Optimal value function	Off-policy
DQN	Model-free	Optimal value function	Off-policy
A3C	Model-free	Optimal policy	On-policy
TRPO	Model-free	Optimal policy	On-policy
PPO	Model-free	Optimal policy	On-policy
DPG	Model-free	Interpolating between policy optimization and optimal value function	Off-policy
DDPG	Model-free	Interpolating between policy optimization and optimal value function	Off-policy

**Table 2:** RL-based research on bionic underwater robots.

Platform	Task	RL Method	Performance
Fish robot (2022) [88]	CPG optimization	DDPG ${}_{Sim,Sim}$	Higher CPG convergence speed
μ Bot (2021) [89]	CPG-based gait learning	PGPE ${}_{Real,Real}$	Speed optimization
Five-jointed robotic fish (2022) [90]	Body-wave-based control	SAC ${}_{Sim,Both}$	Wave parameter optimization
Robotic tadpole (2022) [91]	DMP-based motion control	TRPO ${}_{Sim,Real}$	Validating method effectiveness
Robotic fish (2021) [92]	Bionic control	Q-learning ${}_{Sim,Real}$	Energy saving
Gliding robotic fish (2022) [93]	Bionic gliding control	double DQN ${}_{Sim,Both}$	Energy saving
MT1 Profile (2006) [94]	Bionic control	PG-RL ${}_{Sim,Sim}$	Effective steering
Tail fin (2022) [95]	Bionic flapping motion	On-policy RL ${}_{Real,Real}$	High hydrodynamic efficiency
Beaver-like robot (2022) [96]	Bionic control	Q-learning ${}_{Sim,Real}$	Multiple bionic actions control
Robotic fish (2022) [17]	Attitude holding control	DDPG ${}_{Sim,Real}$	Holding desired angle of attack
Fish-like robot (2022) [18]	Pose, path-following control	A2C ${}_{Sim,Real}$	A general learning framework
Soft robot (2021) [81]	Motion control	SAC ${}_{Sim,Real}$	Line tracking under disturbances
SCP fish robot (2018) [82]	Speed control	Q-learning ${}_{Sim,Sim}$	Effective control method
Robotic eel (2022) [97]	Motion control	SAC ${}_{Sim,Real}$	Effective online control
Fish-like robot (2020) [98]	Path-following control	A2C ${}_{Sim,Real}$	Dynamics-free control
RoboDact (2021) [99]	Yaw, speed control	SAC ${}_{Sim,Real}$	Effective control method
Robotic jellyfish (2019) [100]	Attitude control	Q-learning ${}_{Sim,Real}$	Yaw maneuverability
Soft octopus(2022) [101]	Single-arm attitude control	DQN ${}_{Sim,Real}$	Forward and turning motion
OUC-III (2019) [102]	Attitude control	ADRC + NAC ${}_{Sim,Sim}$	High-precision adaptive control
Bionic manta (2023) [103]	Depth control	Q-learning ${}_{Sim,Real}$	Effective control method
Robotic penguin (2022) [104]	Depth control	MPC-LOS + DDPG ${}_{Real}^{Sim,}$	Effective control method
Soft bionic Pangasius (2022) [105]	Path-following control	**PPO**, A2C, DQN ${}_{Real}^{Real,}$	Effective control method
Bionic vehicle (2022) [106]	Target-following control	DPG-AC ${}_{Sim,Real}$	Effective control method
SCP fish robot (2022) [107]	Yaw, path-following control	DDPG ${}_{Sim,Sim}$	Effective control method
Three-jointed fish robot (2021) [108]	Target-following control	DDPG ${}_{Sim,Real}$	Real-time 2D target tracking
Bionic robotic fish (2021) [109]	Tracking control	DDPG ${}_{Sim,Real}$	Energy-efficient control
Robotic Dolphin (2022) [110]	Path-following control	Improved DDPG ${}_{Sim,Sim}$	Effective control method
Hybrid fish robot (2022) [111]	Path-following control	DDQN ${}_{-,Real}$	Better tracking accuracy
Wire-driven robotic fish (2023) [112]	Path-following control	MARL ${}_{Sim,Real}$	Improved accuracy and stability
Robotic fish (2022) [113]	Speed control	DDPG and **TD3** ${}_{Sim,Real}$	Improved speed tracking
Robotic fish (2022) [114]	Pose control	DDPG-DIR ${}_{Sim,Real}$	Pose control under disturbances
G9 robotic fish (2006) [69]	Underwater searching	Q-learning ${}_{Sim,Real}$	Tank lap swimming
Robotic shark (2022) [87]	Underwater searching	DDPG ${}_{Real,Real}$	Exploration efficiency boost
Self-propelled fish (2021) [115]	Obstacle avoidance	One-step AC ${}_{Sim,Sim}$	Complex obstacles avoidance
Swarm simulator (2018) [116]	Formation control	DDPG+LSTM ${}_{Sim,Sim}$	Formation energy-saving
CFD-based fish (2023) [117]	Formation control	D3QN ${}_{Sim,Sim}$	Leader–follower topology
Fish-like robots (2021) [16]	Formation control	MARL ${}_{Sim,Real}$	Effective circle formation control
Multiple robotic fish (2017) [118]	Coordination control	Fuzzy RL ${}_{-,Real}$	Improving game winning chances
Microswimmers (2022) [119]	Pursue evasion game	NAC + MARL ${}_{Sim,Sim}$	Pursue or evasion decision
Simulated agents (2019) [120]	Leadership decision	PPO ${}_{Sim,Sim}$	Swarm interaction groundwork
RoboDact (2022) [121]	Water Polo Ball Heading	SAC ${}_{Sim,Real}$	Self-heading water polo ball
Underwater robot (2019) [122]	Behavior decision	Q-learning ${}_{Sim,Sim}$	Better decision making

*Sim* and *Real*, respectively, refer to training or deployment conducted in simulated and real environments.

**Table 3:** Existing effective training and deployment frameworks.

ID	Training	Deployment	Primary Computational Cost
1	Simulation-based training	Simulation-based deployment	Robot modeling cost
2	Simulation-based training	Real-world deployment	Robot modeling cost
3 [18]	Numerically driven simulation training ⇒ CFD-based training	Real-world deployment	CFD modeling cost, high-precision simulation cost
4 [131]	Imitation-learning-based teaching ⇒ Simulation-based training	Real-world deployment	Physical data acquisition cost
5 [87]	Imitation-learning-based pre-training ⇒ Real-world training	Real-world deployment	Supervision cost for underwater training, safety risks of robot motion

## Data Availability

The data generated during the current study are available from the corresponding author on reasonable request.
